# Pathogenesis and therapy of radiation enteritis with gut microbiota

**DOI:** 10.3389/fphar.2023.1116558

**Published:** 2023-03-31

**Authors:** Qilin Yang, Bingzhi Qin, Weiliang Hou, Huanlong Qin, Fang Yin

**Affiliations:** ^1^ Research Institute of Intestinal Diseases, Shanghai Tenth People’s Hospital, Tongji University, Shanghai, China; ^2^ School of Clinical Medicine of Nanjing Medical University, Nanjing, China; ^3^ Shanghai Cancer Institute, Renji Hospital School of Medicine, Shanghai Jiao Tong University, Shanghai, China

**Keywords:** radiotherapy, radiation enteritis, pathogenesis, gut microbiota, microbial therapy

## Abstract

Radiotherapy is widely used in clinic due to its good effect for cancer treatment. But radiotherapy of malignant tumors in the abdomen and pelvis is easy to cause radiation enteritis complications. Gastrointestinal tract contains numerous microbes, most of which are mutualistic relationship with the host. Abdominal radiation results in gut microbiota dysbiosis. Microbial therapy can directly target gut microbiota to reverse microbiota dysbiosis, hence relieving intestinal inflammation. In this review, we mainly summarized pathogenesis and novel therapy of the radiation-induced intestinal injury with gut microbiota dysbiosis and envision the opportunities and challenges of radiation enteritis therapy.

## Introduction

**FIGURE 1:**
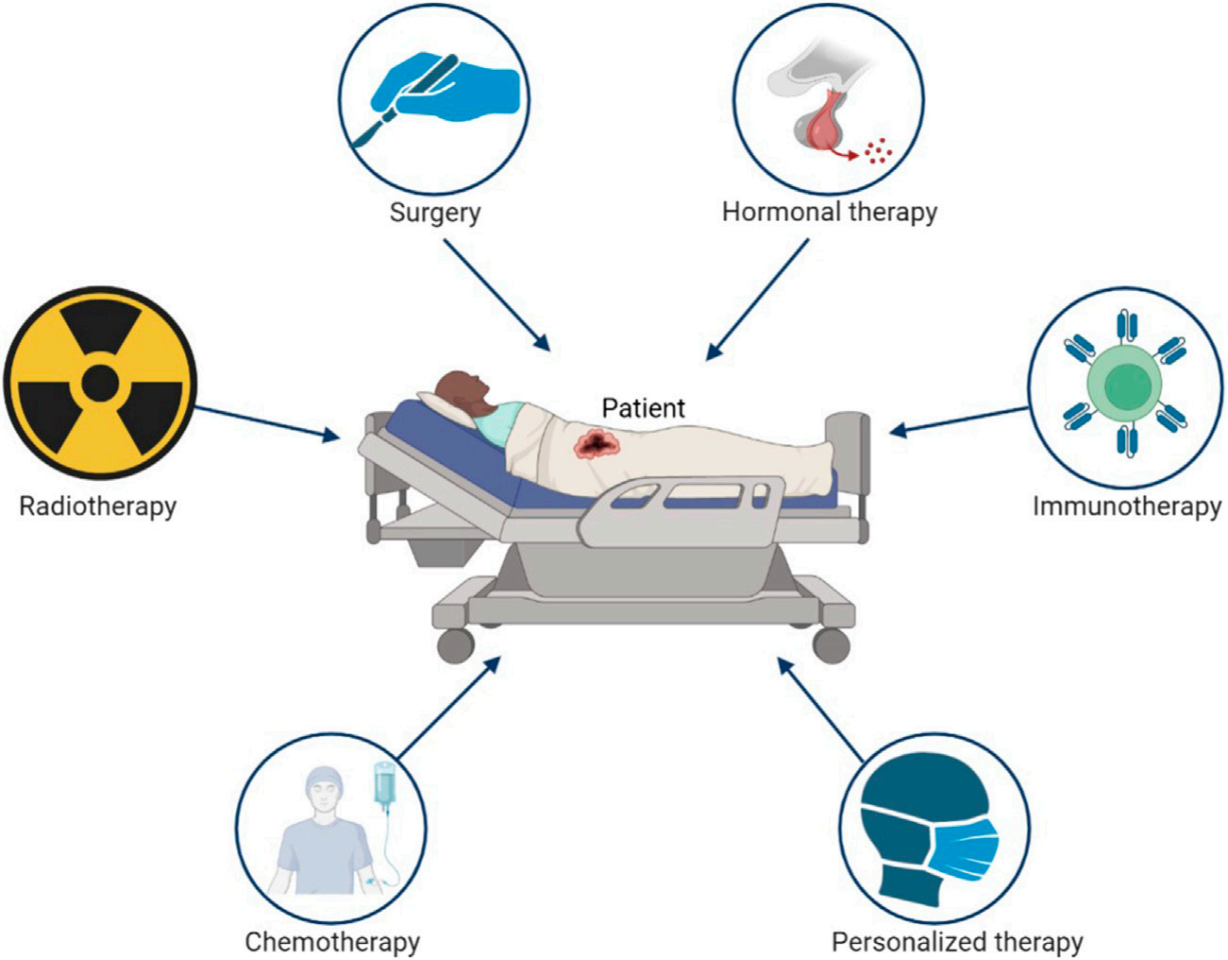
The main treatment strategy of cancer.

With the rapid development of modern medical technology, the average lifetime of human is gradually extended. The global population officially crossed eight billion in the end of 2022. Population aging has become unprecedented phenomenon in human history. The accumulation of exposure to risk factors with aging cause cancer become one of the main death reasons in humans today (Bray et al., 2021), which seriously limits the life span of human. Although the development of science and technology has improved the survival rate of cancer patients, the prevention and treatment of cancer still brings great challenges to human medicine. The emergence of new cancer therapies such as immunotherapy and targeted therapy has made great progress in cancer treatment. However, surgery, chemotherapy, and radiotherapy are still currently the main clinical treatment methods for tumor (Figure 1). It is estimated that more than 70% of tumor patients receive radiotherapy during treatment (Shadad et al., 2013).

Radiotherapy is a local treatment that induces double-stranded DNA breaks leading to tumor cell apoptotic. Tumor cells proliferate rapidly, are sensitive to radiation, and repair more slowly than normal cells, so they have good therapeutic effects on many types of tumors. Radiation also damages normal cells and tissues, especially active proliferation cells, which are highly sensitive to irradiation and very vulnerable to damage. Therefore, radiotherapy for many cancers can lead to many adverse reactions, such as radiation dermatitis in breast cancer and pulmonary fibrosis in lung cancer (Werner-Wasik et al., 2004; Lewis and Farach, 2019; Ramseier et al., 2020). Meanwhile, many side effects are also different due to the different exposure position of radiotherapy. Gastrointestinal cells are sensitive to ionizing radiation, which limits the radiation treatment of malignant tumors in the abdomen and pelvis. Radiotherapy of malignant tumors in the abdomen and pelvis is easy to lead to anorexia, nausea, vomiting, bloody stools, diarrhea, mucous stools and other complications of radiation enteritis, which brings a great burden to patients physically, psychologically and economically (Bismar and Sinicrope, 2002).

The human microbiota contains 10^13^–10^14^ cells with a ratio of about 1:1 to human cells (Sender et al., 2016). Studies suggested that the bacteria species that colonize each individual is more than 800 (Turnbaugh et al., 2010). In individuals, the gut microbiota maintained a mutualistic symbiotic relationship with the host. The gastrointestinal microbiota was mainly composed of bacteria from three major phyla, *Firmicutes*, *Bacteroidetes*, and *Actinobacteria* (Tap et al., 2009). The microbiota change affects the health status of the host, and the health status of the host also can lead to the microbiota imbalance, which was found in a variety of diseases such as inflammatory bowel disease, Parkinson’s disease, diabetes, hypertension, depression, colorectal cancer and so on (Kho and Lal, 2018). Irradiation exposure can cause the microbiota dysregulation, resulting in a relative decrease in probiotics (such as *Bifidobacterium* and *Lactobacillus*) and a parallel increase in harmful or even pathogenic microorganisms (such as *Fusobacterium* and *Proteus*) (Lam et al., 2012; Jian et al., 2021).

Radiation-induced gastrointestinal injury urgently needs effective therapy. People have obtained a lot of achievements by exploring novel therapy, such as drug therapy, stem cell therapy and microbial therapy. However, there has been no widely recognized strategy on the treatment of radiation enteritis at present. Herein, we mainly reviewed pathogenesis and treatment of radiation enteritis with gut microbiota dysbiosis and envisioned the opportunities and challenges of radiation enteritis therapy.

## Pathogensis of radiation enteritis

### The normal intestine maintains homeostasis

The gut is not only the digestive organ of human, but also the largest endocrine and immune organ in the body, responsible for digestion and absorption, immunologic barrier and food residue transport. The integrity of intestinal morphology is the premise and basis of intestinal normal functions. The damage of intestinal integrity can affect the digestion, absorption and transportation, hence leading to the destruction of mucosal barrier functions. The incomplete mucosal barrier permits intestinal bacteria and toxins into the systemic circulation, resulting in the release of a large number of inflammatory cytokines, oxygen free radicals and other harmful substances, and eventually damaging multiple organs.

**FIGURE 2:**
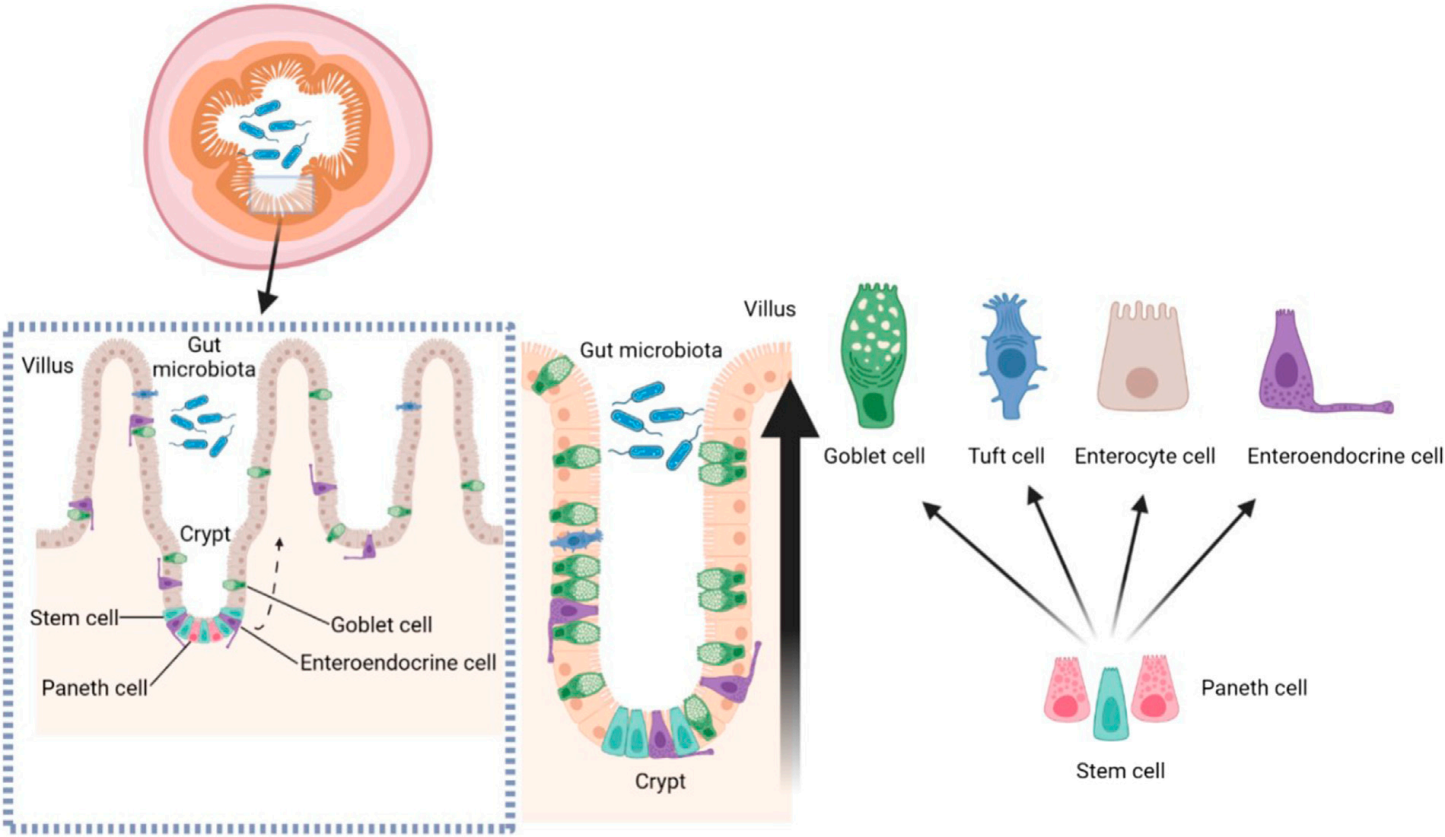
The structure of intestinal crypt-villus.

Villi are finger-like protrusions on the small intestine that are essential for nutrient absorption. The disruption of villus structure is bound to affect intestinal health. Crypts are invaginations of perivillous epithelial cells with continuously dividing stem cells at the base. These stem cells continuously divide and provide the source of all other epithelial cells. Briefly, stem cells in the crypts maintain the structure of the villi (Clevers, 2013). Leucine-rich repeat-containing G-protein coupled receptor five positive (LGR5+) stem cells are positioned at the bottom of the crypt, mixed with Paneth cells in the small intestine. In order to maintain the epithelial barrier, intestinal stem cells divide asymmetrically at the crypt bottom and generate new stem cells and daughter cells, which differentiates towards these cells of the secretory and absorptive lineage along the crypt-villus axis (Ruder et al., 2019). The differentiated cells (except Pan’s cells) gradually mature as they move up the crypt-villus axis, finally reaching to the apical region of the villus (Figure 2). Under normal circumstances, intestinal mucosal cells can rapidly self-renewed within 5 days in human body. Intestinal mucosal cells can present proliferation, differentiation, damage repair and apoptosis simultaneously (Ramadan et al., 2022). The homeostasis of normal intestine has been explained to be dependent on mitochondrial function: Undifferentiated stem cells use glycolysis to generate ATP, whereas along crypt-villus axis, ATP production shifts from glycolysis to mitochondrial oxidative phosphorylation. And the mitochondrial electron transport chain is regarded as the major source of mitochondrial reactive oxygen species (ROS). In general, differentiation of stem cells is often accompanied by increased oxygen consumption and ROS levels. Furthermore, activity in terms of oxidative phosphorylation and ROS formation was associated with activation of p38. Hence, mitochondrial function has profound roles in all aspects of intestinal homeostasis (Rath et al., 2018). A variety of signaling molecules play crucial roles in maintaining intestinal homeostasis, such as P53, P21, Bax, Bcl-2, CD95 and so on (Anwar et al., 2017). In addition, Wnt, BMP, Hedgehog, PTEN/PI3K and Notch signaling pathways also play vital roles in the self-renewal of intestinal stem cells, ensuring the integrity of intestinal morphology and structure (Meena et al., 2022). The digestive tract has a surface area of over 30 m and is primarily structured by villi for better absorption of nutrients (Helander and Fändriks, 2014). Intestinal stem cells are present in the villi crypt. The destruction of the villi structure affects the proliferation and differentiation function of the stem cells and prevents the formation of intestinal epithelial cells. Disruption of the intestinal barrier leads to bacterial translocation and a reduction in the intestinal surface area affects the absorption of nutrients and water. Damage to the intestinal villi may even cause repeated replication and proliferation of intestinal stem cells, increasing the risk of DNA damage and thus malignancy (Gehart and Clevers, 2019).

The gastrointestinal tract is home to an enormous number of microbes which regulate the physiological and pathological processes of theirs host, and thus normal homeostasis encompasses the dynamic stability of the microbiota. The intestinal microorganisms play a key role in nutrient absorption, vitamin synthesis, inflammatory regulation, and host immune response. For instance, gut microbiota is an important source of essential nutrients, including B-vitamins (Magnúsdóttir et al., 2015). Hyperlipidemia is associated with change of the abundance of specific bacterial taxa short chain fatty acid-producing bacteria, such as the families *Lachnospiraceae* and *Ruminococcaceae* and the genera *Akkermansia*, *Bacteroides*, *Roseburia*, and *Faecalibacterium* (Gargari et al., 2018). Intestinal microbiota influences vitamin D distribution and metabolism (Huang et al., 2019). Stimulator of interferon genes promotes intestinal immunoglobulin A by regulating acetate-producing gut bacteria (Yu et al., 2023).

### Irradiation damages intestinal integrity

**FIGURE 3:**
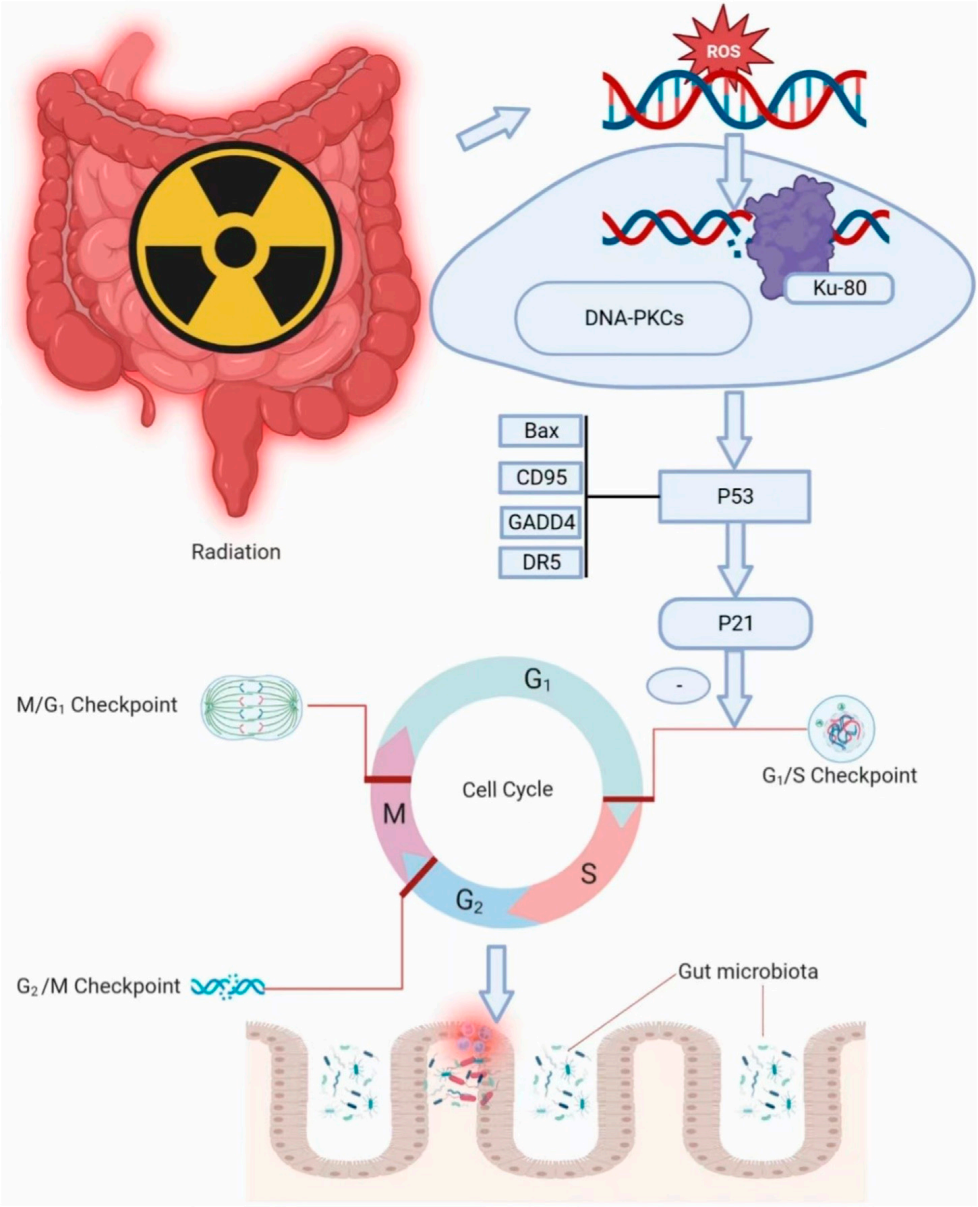
Irradiation damages intestinal integrity.

Intestinal damage from radiation therapy leads to gastrointestinal reactions in the first or second week, with a few occurring several hours after radiation. The intestinal damage is often reversible caused by low doses of radiation. 40% of patients can accompany with significant lesions at the radiation dosage of 10–30 Gy, while the number will sharply increase to 90% with the dosage beyond to 30 Gy (Bismar and Sinicrope, 2002). Radiation enteritis consists of five stages, including the initial stage, the major damage response stage of inflammation and apoptosis, the signal amplification stage, the ulceration stage and the healing stage (Sonis, 2004). Radiation effects can directly or indirectly impact on cellular DNA (Bismar and Sinicrope, 2002). In response to the DNA damage, most of the cells prevents cell progression entry into the S phase in the late G1 to arrest cell cycle. The ROS directly affect DNA molecules and lead to the breakage of single-strand or double-strand (Morgan and Lawrence, 2015). Ku-80 binds to the broken DNA strand and then binds to inactive enzymes called DNA-dependent protein kinases. DNA-dependent protein kinases binding activates kinase activity, leading to phosphorylation of substrate molecules that activate p53 (Sakaguchi et al., 1998). Activation of p53 stimulates the transcription of p21, which is a cdk inhibitor that binds to G1/S-cdk and S cdk complexes to block the cell cycle progression through the S phase (Bartek and Lukas, 2001). A reduction in the number of mitotic cells due to cell cycle arrest, and some cell death without cell proliferation contribute to the sterilization of many, but not all, crypts, followed by severe depletion of villus cells. Villi cells depletion exposes animals to the infectious contents of the gut and causes loss of the fluid barrier, which can largely contribute to cell death (Anwar et al., 2017) (Figure 3). In addition, DNA damage activates the related signaling pathways (such as P53 and NF-κB), and releases the proinflammatory factors (such as TNF-α, IL-6, IL-1β), which can lead to apoptosis (Criswell et al., 2003). In addition, the activation of glycolytic pathway and Fas cell signaling pathway are also related to radiation-induced apoptosis (Song et al., 2014). RNA and protein molecules in the exosomes of irradiated cells can initiate non-targeted effects in a synergistic manner, acting on non-irradiated cells and causing chromosome instability (Al-Mayah et al., 2015; Tomita and Maeda, 2015). Irradiation destroys the dynamic balance of proliferation and differentiation of crypt stem cells, hence leading to intestinal villi damage, discontinuity of epithelial barrier, permeability change and microbiota translocation (Potten et al., 2002; Sonis et al., 2004).

The intact gut acts as the barrier function to prevent damage from microorganisms and their metabolites. The intestinal barrier is composed of intestinal epithelial cells, lamina propria, mucus layer and intestinal microbiota (Takiishi et al., 2017). Epithelial cells and lamina propria provide physical barriers to prevent material leakage from the intestinal lumen and work in coordination with immune cells and stromal cells to limit their direct contact with the epithelium and repel pathogens (Odenwald and Turner, 2017). Monolayer epithelial cells and tight junctions (TJ) maintain the stability of the intestinal environment by controlling paracellular permeability (Schulzke et al., 2009). The integrity of intestinal epithelium is easy to be impaired after irradiation (Shukla et al., 2016). The intestinal mucus layer protects intestinal cells exposure to external and toxic substances, digestive enzymes and bacteria, hence playing a major role in intestinal defense against mechanical, chemical and biological attacks (Etienne-Mesmin et al., 2019). Irradiation decreased the number of goblet cells and the expression of mucin 2 (MUC2) (Paone and Cani, 2020; Jang et al., 2022).

Radiation reduced the number of microvessels and capillary density (Lam et al., 2012). Signal molecules play an important role in vasodilation and vasoconstriction, such as nitric oxide (NO), prostacyclin and hydrogen peroxide derived from vascular endothelial cells. After radiation, the bioavailability decrease of vascular endothelial cells leads to the enhanced vascular tone, vascular dysfunction, upregulation of adhesion molecules expression, and promotion of platelet aggregation (Stewart et al., 2010). The generated ROS activates the NF-κB pathway, hence increasing expression of TNF-α, IL-1β, IL-6, etc., which plays a vital role in the regulation of inflammation. Radiation can also cause chronic ischemia, intestinal fibrosis and high expression of vascular endothelial growth factor (VEGF) (Kawano et al., 2006; Beenken and Mohammadi, 2009; Stansborough et al., 2018).

Microbiota is the most active element in the intestinal barrier, which can adhere to the intestinal mucosa to form the additional protective layers (Pickard et al., 2017). At the same time, the composition and species of intestinal microbiota remains relatively stable under normal state. Dynamically balanced homeostasis of normal gut microbiota plays an irreplaceable role in protecting intestinal integrity. Pathogenic bacteria interact with Toll-like receptors and subsequently activate NF-κB signaling pathway, compounding the inflammatory response through the generation and amplification of TNF-α, IL-1β, and IL-6 (Stringer, 2013). Corresponding to the pathogenic, many commensal microbiotas can decrease NF-κB activation, such as *Bifidobacterium infantis*, *Bacteroides thetaiotaomicron*, and *Faecalibacterium prausnitzii*, (Kelly et al., 2004; Ewaschuk et al., 2008; Sokol et al., 2008). By occupying the epithelial surface, gut microbiota prevent the adhesion of harmful bacteria to produce antimicrobial substances, hence jointly maintaining the integrity of the intestinal barrier (Iacob et al., 2018). Increased radiation toxicity is associated with decreased gut microbiome diversity (Mitra et al., 2020). Irradiation causes the dysregulation of gut microbiota, including the decreased abundance of beneficial bacteria and the increased abundance of pathogens (Zhao T. S. et al., 2021). Patients receiving radiotherapy showed prominent changes in gut microbiota, with most frequently, decrease in *Bifidobacterium*, *Clostridium* cluster XIVa, *Faecalibacterium prausnitzii*, and increase in *Enterobacteriaceae* and *Bacteroides* (Touchefeu et al., 2014). When the number of probiotics decreases, conditional pathogens will multiply occupy the ecological niche, hence inhibiting the growth of probiotics and promoting the release of endotoxin (Jian et al., 2021).

## Diagnose and precaution of radiation entiritis

### Clinical manifestations of radiation enteritis

Patients present the related symptoms after one to 2 weeks of radiotherapy. The upper abdominal radiotherapy impacts on small intestine and stomach causing stomach cramps, short-term diarrhea and nausea. The lower abdominal irradiation involves large intestine contributing to tenesmus, diarrhea and rectal bleeding (Porouhan et al., 2019). The clinical manifestations of acute radiation enteritis present nausea, vomiting, abdominal pain, diarrhea, increased fecal frequency, mucous, pus and blood stool, and even death in severe cases. The clinical manifestations of chronic radiation enteritis involved chronic intestinal obstruction, intestinal perforation, fistula and abscess, etc. (Loge et al., 2020).

### Diagnose of radiation enteritis

Clinical diagnosis of radiation enteritis is mainly based on the history of radiation combined with clinical manifestations and imaging findings of enteritis (Porouhan et al., 2019). For example, X-ray angiography can show small intestinal ring separation and mucosal edema in the intestinal lumen (Tabaja and Sidani, 2018). Colonoscopy can confirm the location of stenosis and bleeding. Computer Tomography (CT) scan or Magnetic Resonance Imaging (Kroemer et al., 2009) can determine the location of obstruction and evaluate intestinal involvement (Porouhan et al., 2019).

The disorder of intestinal flora is one of the characteristics of radiation enteritis, so the detection of intestinal flora is also a reference for diagnostic criteria. 16S rRNA sequencing is the most commonly used method to study the distribution and diversity of gut microbiota among individuals (Liu et al., 2021). In many studies, 16S rRNA sequencing has detected changes in the abundance of bacteria in radiation enteritis (Kim et al., 2015).

### Precaution of radiation enteritis

Radiation dose is a key factor for the occurrence of radiotoxicity. High dose better killed tumor cells, but significantly increased the death of normal cells (Vargas et al., 2005). The extent of intestinal damage depends on the physical characteristics of radiation exposure, including dose rate, treatment dose segmentation, total dose, field size, and radiation type. Particularly, the total dose and time of duration of radiation exposure might have a significant influence on the results of changing in composition, richness, and diversity of the gut microbiota (Fernandes et al., 2021).

The radiotherapy equipment determines the technical level of radiotherapy. Advanced instruments can provide a good irradiation strategy to improve the irradiation efficiency, reduce the damage to normal tissues and alleviate the effect of irradiation on intestinal flora. Radiotherapy is currently divided into conventional radiotherapy, three-dimensional conformal radiotherapy, intensity modulated radiotherapy, image-guided radiotherapy, and dose-guided radiotherapy. Image-guided radiotherapy based on CT, Cone Beam CT (CBCT), or MRI can better target the tumor and the risky area, hence preserving the nearby normal tissue and providing more options for treatment (You and Hou, 2022). Magnetic resonance image-guided radiation therapy system constructed by MRI can better describe soft tissue, especially for the observation of perineural infiltration, extracapsular extension and muscle infiltration. This therapy system shows very low gastrointestinal and genitourinary system radiation toxicity in the radiotherapy of prostate cancer (Bruynzeel et al., 2019). Moreover, the combination of the new technology with radiotherapy hardly increases the risk of side effects from additional irradiation. For example, the estimated dose per CBCT is only 0.03 Gy. For each patient, the additional radiation dose from radical radiotherapy is less than 1 Gy (Kong et al., 2021). The impact of radiation on normal tissues can be reduced by the lead plate to block the visual field outside the tumor. Besides, laparoscopic tissue expander inserted into the lower pelvis for removing the intestinal tract out of the radiation field, which can effectively prevent radiation enteritis (Omejc et al., 2021). Lattice Radiation Therapy is a spatially fractionated radiotherapy technique that allows simultaneously deliver sufficient ablation dose to the inside neoplastic lesions and low dose to adjacent organs (Iori et al., 2023).

The formulation of individualized radiotherapy strategy and preoperative determination of the response of colorectal cancer patients to chemotherapy or chemoradiotherapy can effectively control the radiation dose, thereby reducing the toxic side effects of radiation and the high-risk factors. Among them, organoids are great potential to develop personalized treatment plans, which can simulate different developmental stages and disease states (Suarez-Martinez et al., 2022). Studies have used patient-derived organoids to evaluate the response of patients to radiation for developing the personalized radiotherapy strategies (Hsu et al., 2022). According to some studies, the underlying diseases and poor living habits can increase the sensibility for radiation toxicity. For example, diabetes has been proved to be an independent risk factor for late irradiation damage. The incidence of radiation enteritis in diabetic patients is higher than that in non-diabetic patients (Herold et al., 1999). The complications of radiation enteritis in patients with inflammatory bowel disease may be more severe (Tromp and Christie, 2015). Smoking may aggravate the gastrointestinal toxicity from radiation (Wedlake et al., 2010). Therefore, the treatment of underlying diseases and good health habits may reduce the incidence and complications of radiation enteritis.

Before irradiation, the administration of radiation protective agent can reduce the radiation toxicity of normal cells and effectively kill tumor cells. The first clinical radiation protective agent approved by the Food and Drug Administration is amifostine. The administration of amifostine within 15–30 min before radiotherapy can effectively protect the normal tissues of radiation toxicity (Tas et al., 2016; King et al., 2020). However, its oral application for clinical radiation protection remains challenging. Therefore (Zhang et al., 2022), constructed an oral delivery system for amifostine using the microalga *Spirulina platensis*, which was significantly superior to the free drug and its enteric-coated capsules. CBLB502 is a kind of radiation protective agent derived from bacteria, a polypeptide drug derived from *Salmonella* flagellin, can bind to Toll-like receptor five and activate NF-κB signaling (Burdelya et al., 2008). Polydopamine nanoparticles gavage before irradiation protected mice from irradiation-induced crypt villus unit damage, inhibited the depletion of LGR5+ intestinal stem cells (ISCs), promoted cell regeneration, significantly inhibited intestinal cell apoptosis, inflammatory necrosis and DNA damage (Jia et al., 2022). Vitamin D prevents irradiation-induced intestinal injury by inhibiting crypt trunk/progenitor cell apoptosis mediated by PMAIP (Phorbol-Myristate-Acetate-Induced Protein) (Li W. et al., 2021). The combination of fibroblast growth factor 1 (FGF1) with high-sulfated hyaluronic acid (HA-HS) and heparin (HP) before irradiation could increase the survival rate of the crypt. HA-HS better presented weak anticoagulant effect and reduced the risk of intestinal bleeding compared with HP, which can be a better choice for irradiation precaution (Miura et al., 2022).

In addition, some compounds also can reduce the intestinal damage by radiation (Wang et al., 2022b). The administration of radiosensitizer before irradiation can increase the effect of irradiation and thereby reduce the radiation dose, hence weakening the side effects of irradiation. The compound ((E)-5-(2-([1,1′-biphenyl]-4-yl) vinyl)-2-hydroxybenzoic acid, DC10) acted as a radiosensitizer by inhibiting cystine uptake and increasing oxidative stress (Sarowar et al., 2022). SET domain containing (lysine methyltransferase) eight inhibition potentiates carcinomas radiotherapy by enhancing radiosensitivity to suppress DNA damage repair of tumor cells (Pan et al., 2022). Thioredoxin reductase (TrxR) is an operator of several cellular processes. Tumor cells elevate the level of TrxR, hence contributing to tumorigenesis and angiogenesis in the early stages, and metastasis and radioresistance in late stages of cancer. Therefore, TrxR inhibitors have great potential as radiosensitizers, but the relevant clinical effect needs to be verified (Patwardhan et al., 2022). Since the main killing process of tumor cells by radiotherapy is the use of ROS, the hypoxia environment may be a key factor affecting the radiation dose. Therefore, many researchers have developed a number of nanomaterials targeting to change the hypoxia environment in tumors, hoping to reduce the relative dose of radiotherapy and reduce the side effects of irradiation (Li et al., 2018).

### Therapeutic strategy

Nowadays, there are not recognized effective treatment for radiation enteritis. However, symptomatic treatment, drug therapy, stem cells therapy, organ transplantation and microbial therapy have showed good clinical prospects.

### Symptomatic treatment of radiation enteritis

The symptoms of radiation enteritis include diarrhea, nausea, vomiting, stomach cramps, fecal urgency, loss of appetite, pain after eating, acute or intermittent small bowel obstruction, nausea, loss of appetite, weight loss, bloating, diarrhea, etc. Symptomatic treatment for clinical manifestations is an effective way, including antidiarrheal, fasting, parenteral nutrition, etc.

Diarrhea is a common clinical manifestation of radiation enteritis. The incidence of chemotherapy-induced diarrhea has been reported as high as 50%–80% of patients (Touchefeu et al., 2014). Treatment of diarrhea can better control enteritis and improve the life quality of patients. Montmorillonite powder, a clinically effective drug for the diarrhea treatment, combined with dexamethasone is effective in treating acute radiation enteritis, which can significantly alleviate mucosal damage, improve inflammation, and promote patient recovery (Chen et al., 2021). TGF-β2 enriched formula supplemented with nutrition can improve radiation-induced diarrhea for patients receiving pelvic radiotherapy (Demiral et al., 2015). Dietary probiotics and fecal microbiota transplantation (FMT) were widely applicated to treat or prevent diarrhea (Li Y. et al., 2021). Probiotics have been proved in some clinical studies that they significantly reduced the incidence of severe diarrhea induced by radiation therapy, such as VSL#3 formulation containing *Bifidobacterium, Lactobacillus* and *Streptococcus* (Delia et al., 2007). Intestinal obstruction is one of the symptoms of radiation enteritis, so fasting for patients with this complication has certain benefits. Several researchers have demonstrated this in animal experiments, where fasting can profitably improve the regeneration of intestinal stem cells and the survival rate of mice (de la Cruz Bonilla et al., 2019). In patients with radiation enteritis, absorption dysfunction causes nutritional deficiency. Therefore, nutritional therapy becomes an effective option. Nutrition therapy is divided into enteral nutrition and parenteral nutrition, both of which have their own characteristics and advantages. Enteral nutrition plays an important role in the recovery of inflammatory bowel disease and is often the first choice of nutritional therapy because it is non-invasive and can help to restore intestinal function (Luo J. et al., 2022). However, parenteral nutrition becomes the first choice when radiation enteritis is accompanied by intestinal obstruction or when fasting is required before surgery (Hellerman Itzhaki and Singer, 2020). Regardless of parenteral nutrition or enteral nutrition, the addition of certain nutrients to the nutrient solution may contribute to the recovery of the condition, for example, Glutamine can provide essential nutrition for the intestine, prevent intestinal mucosal atrophy and ameliorate intestinal immune function (Scolapio et al., 2002). For patients with pain, analgesia may be a necessary choice after the cause of the disease is identified, and it has an irreplaceable effect on the condition. Opioid analgesics have dual functions of analgesia and intestinal motility reduction in the treatment of radiation enteritis (Hale, 2020).

When patients have serious complications, surgical treatment has become an important treatment method, such as intestinal perforation, intestinal obstruction, intestinal fistula and massive intestinal bleeding. About one-third of patients with chronic radiation enteritis require surgical treatment (Huang et al., 2016). In the treatment of intestinal strangulation caused by chronic radiation enteritis, laparoscopic surgery can reduce incision length and blood loss (Wang et al., 2015). In addition, endoscopy can be used to treat bleeding spots in easily accessible areas of the colon or small intestine. Surgical treatment is a feasible plan for perforation and intestinal strangulation caused by radiation enteritis, but preoperative preparation should be done to reduce mortality rate and surgery complications.

### Drug therapy of radiation enteritis

**FIGURE 4:**
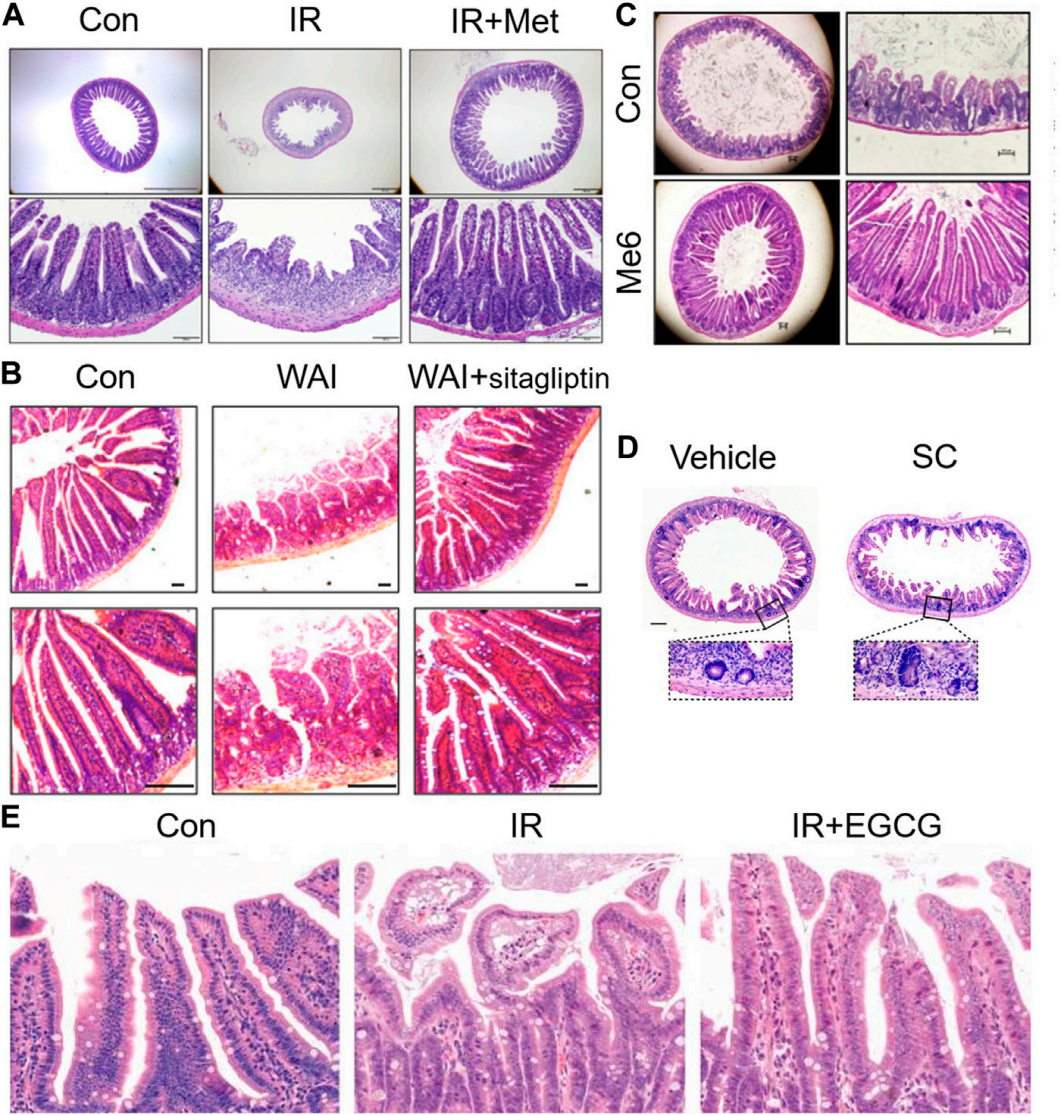
Therapeutic effects of drugs on radiation enteritis. **(A)**. Metformin increased villus length and crypt number after irradiation. **(B)**. Sitagliptin reduced the intestinal damage caused by irradiation in mice. **(C)**. Me6TREN promoted intestinal tissue regeneration after radiation injury. **(D)**. CBP/P300 inhibitiors promoted the regeneration of crypts *in vivo*. **(E)**. EGCG administration reduces radiation-induced intestinal mucosal injury significantly by increasing the number of LGR5 + ISCs and Ki67 + crypt cells.

Radiation enteritis caused by radiation causes DNA damage through ROS produced by ionization, which activates P53 and other signaling pathways, causes abnormal apoptosis of intestinal stem cells, destroys the structure and function, and causes intestinal flora disorder. Therefore, according to its pathological process, many compounds and drugs have been proposed for the treatments of radiation enteritis, and good results have been achieved. Aminosalicylate is commonly used in inflammatory bowel disease, but its efficacy in the treatment of radiation enteritis is uncertain. Studies have shown that aminosalicylate may worsen gastrointestinal symptoms (such as diarrhea or rectal bleeding), hence increasing the additional medical treatment (Lawrie et al., 2018). Metformin is the most widely used antidiabetic drug in clinic, and presents anti-inflammatory, antioxidant and anti-apoptotic effects (Xue et al., 2019). The damage of crypt stem cells and intestinal barrier were the most common changes caused by the hypersensitivity of tight junctions (TJ) to radiation. Studies have found that metformin increased villus length and crypt number by activating Wnt/β-catenin signaling pathway, increased tight junction expression of epithelial cells, inhibited bacterial metastasis to mesenteric lymph nodes, and restored intestinal barrier function of irradiated intestine (Jang et al., 2022) (Figure 4A). Vitamin D protected the intestinal barrier and altered the gut microbiota such as markedly decreasing typical conditional pathogen species including *Pseudomonas*, *Escherichia*, and *Shigella* (Huang et al., 2019). Supplementation of antioxidants vitamins A, C, E or lycopenes reduced the ROS from irradiation and protected the epithelial cell and restored their absorptive functions to patients undergoing their treatment. Vitamin E supplementation, in particular, compared to vitamins A and C, proved to be most effective in ameliorating the activities of intestinal digestive enzymes (Anwar et al., 2013). FG-4592, a novel up-regulator of hypoxia Inducible factor, could remit radiation enteritis by promoting regeneration and differentiation of intestinal stem cells (Xia et al., 2022). The CBP and p300 protein family consists of cyclic-AMP response element-binding protein-binding protein (CBP) and E1A-binding protein (P300). CBP/P300 inhibitors promoted the regeneration of intestinal organoids *in vitro* and remitted radiation-induced gastrointestinal syndrome by delaying intestinal epithelial cell cycle progression (Rao et al., 2021) (Figure 4). Sirtuin one inhibitors could be effective clinical countermeasures to mitigate gastrointestinal toxicity by increasing p53 acetylation and the stabilization of p53, and likely contributing to the survival of intestinal epithelial cells in post-radiation (Fu et al., 2021). The small molecule tris [2-(dimethylamino) ethyl] amine induced intestinal stem cells proliferation, enhanced intestinal organoid growth *in vitro*, and promoted intestinal tissue regeneration by activating *β*-catenin signaling after radiation injury (Wang et al., 2020). Sitagliptin, a dipeptidyl peptidase IV inhibitor, reduced NOD-like receptor thermal protein domain associated protein 3 (NLRP3) inflammasome by upregulating Nrf2/NLRP3 pathway to reduce the intestinal damage and significantly recovered gut microbiota balance (Figure 4B). Therefore, sitagliptin may be a feasible treatment strategy for radiation enteritis (Huang et al., 2022). Supplementation of nicotinamide mononucleotide alleviated intestinal fibrosis and ameliorated radiation-induced intestinal microbiota disorder and dysfunction (Zhao et al., 2022). Sirtuin 1 inhibitors could be effective clinical countermeasures to mitigate gastrointestinal toxicity by increasing p53 acetylation and the stabilization of p53, and likely contributing to the survival of intestinal epithelial cells in post-radiation (Fu et al., 2021). The small molecule tris [2-(dimethylamino) ethyl] amine induced intestinal stem cells proliferation, enhanced intestinal organoid growth *in vitro*, and promoted intestinal tissue regeneration by activating *β*-catenin signaling after radiation injury (Wang et al., 2020) (Figure 4C). FG-4592, a novel up-regulator of hypoxia Inducible factor, could remit radiation enteritis by promoting regeneration and differentiation of intestinal stem cells (Xia et al., 2022). The CBP and p300 protein family consists of cyclic-AMP response element-binding protein-binding protein (CBP) and E1A-binding protein (P300). CBP/P300 inhibitors promoted the regeneration of intestinal organoids *in vitro* and remitted radiation-induced gastrointestinal syndrome by delaying intestinal epithelial cell cycle progression (Rao et al., 2021) (Figure 4D). The green tea polyphenol (−)-epigallocatechin-3-gallate administration significantly reduced radiation-induced intestinal mucosal injury by increasing the number of LGR5+ISCs and Ki67+ crypt cells, reversing radiation-induced gut dysbiosis, restoring the Firmicutes/Bacteroidetes ratio, and increasing the abundance of beneficial bacteria (Cai et al., 2022) (Figure 4E). Disulfide can regulate DNA damage response and survival of ISCs by affecting cell cycle (Yuan et al., 2022). Paeoniflorin, a pinane monoterpene bitter glycoside, could increase Suppressor of cytokine signaling 3 (SOCS3) expression, active the Gas6/Axl/SOCS3 axis and subsequent reduction in intestinal inflammation and ischemia (Sheng et al., 2022).

### Stem cells therapy of radiation enteritis

Cells with high proliferative potential such as hematopoietic stem cells and ISC are the main targets of radiation-induced injury. Moreover, stem cell transplantation and gut microbiota have been shown to be mutually promoting and synergistic (Zhao L. et al., 2021; Ahmed and Massri, 2022; Tu et al., 2022). Therefore, stem cell transplantation may be a feasible treatment for radiation-induced injury. LGR5+ rectal stem cells are sensitive to radiation and participate in radiation-induced rectal epithelial toxicity. Transplantation of LGR5+ rectal stem cells can alleviate radiation-induced rectal injury and promote the process of rectal repair and regeneration (Tirado et al., 2021).

Mesenchymal stem cells (MSCs) are a class of pluripotent stem cells with self-renewal and multidirectional differentiation properties, which are widely used in clinical treatment of various diseases (Sun et al., 2022). MSCs can home to tissue injury induced differentiation and form specific cell types (Guillamat-Prats, 2021; Liang et al., 2021). Wharton’s jelly-derived MSCs can home to the damaged tissues in irradiated host and mitigate radiation-induced damage for radiosensitive tissues such as hematopoietic and gastrointestinal systems (Bandekar et al., 2021). Bone marrow-derived MSCs can induce the regeneration of intestinal epithelial cells, regulate the secretion of serum cytokines and the expression of radioprotective proteins (Xu et al., 2016). Epithelial injury and infiltration of inflammatory cells in the rectum were significantly suppressed by transplantation of human amnion-derived MSCs (AMSCs), possibly through inhibition of cell injury and inflammatory reactions (Ono et al., 2015). Besides, MSCS can also have a significant impact on the composition of gut microbiota. The oral delivery of stem-cell-loaded hydrogel microcapsules ameliorated the dysbiosis of specific bacterial genera, including *Bacteroides acidifaciens, Lactobacillus (L.) gasseri, Lactobacillus reuteri,* and *L. intestinalis* (Kim et al., 2022). MSCs transplantation could be considered as a new treatment for radiation proctitis.

It has also been suggested that the regenerative effect of MSCs is not dependent on their ability to differentiate and replace damaged tissues, but is mainly mediated by paracrine released factors, including extracellular vesicles composed of microvesicles and exosomes (Aghajani Nargesi et al., 2017). MSCs exosomes are nanosized vesicles secreted by mesenchymal stem cells that retain the therapeutic properties of the cells, including genetic material, lipids, and proteins (Lee et al., 2021). For example, MSCs exosomes reshaped the intestinal microbiota composition by significantly increasing the abundance of probiotics, decreasing disease-associated bacteria (Ocansey et al., 2022). MSCs promoted the secretion of exosomal MicroRNA-34a-5p and improved intestinal barrier function through Methyltransferase-like 3/insulin-like growth factor 2 mRNA binding protein 3-mediated pre-miR-34 A m^6^A modification (Li et al., 2022). Intravenous infusion of human embryonic stem cells alleviated colitis in mice by increasing circulating insulin-like growth factor 1 levels and maintaining the integrity of epithelial cells and promoting their repair and regeneration (Xu et al., 2020).

### Bone marrow transplantation and organoid transplantation

Bone marrow transplantation (BMT) is a special therapy to treat diseases such as leukemias, lymphomas, aplastic anemia, immune deficiency disorders, and some solid tumor cancer. BMT triggered trafficking of host CD11b (+) myelomonocytic cells from host marrow by increasing VEGF, basic FGF (bFGF) and other angiogenic and chemotactic cytokines, enhancing the proliferation of intestinal stroma cells, secondarily leading to epithelial regeneration in the radiation-injured intestinal mucosa (Chang et al., 2012). Parenteral insulin-like growth factor 2 synergized with BMT to confer potent mitigation against radiation-induced gastrointestinal syndrome (Kim et al., 2018). BMT increased blood levels of intestinal growth factors and induced regeneration of the irradiated host ISC niche (Saha et al., 2011). However, graft-versus-host disease after BMT may increase the risk of dysbiosis. Therefore, regulating gut flora before BMT may mitigate inflammation and improve outcomes for BMT (Jenq et al., 2012). Therefore, bone marrow transplantation is also a potential treatment for radiation enteritis.

Organoids are 3D cell cultures derived from stem cells and differentiated into key properties of related organs by adding specific factors. To some extent, the emergence of organoids has made up for the shortcomings that two-dimensional cell lines cannot fully summarize the characteristics of real organisms and animal models and simulate the cell niche in patients. Intestinal organoids have been shown to contain functional intestinal epithelial cells, goblet cells, pan cells, and neuroendocrine cells (Spence et al., 2011). Organoids have been far superior to normal two-dimensional cell cultures, but they are still imperfect. For example, the structure of organoids does not contain endothelial cells, immune cells and intestinal microbes (Luo H. et al., 2022). However, some researchers still studied cell-to-cell and cell-to-microbial interactions by injecting fecal microbes into colon organoids *via* microinjection (Nigro et al., 2019). Organoids has become a promising method in drug discovery, testing and personalized medicine (Suarez-Martinez et al., 2022). Therefore, it has been used in intestinal radiation studies, and some studies have used patient-derived organoids as a means of pre-evaluating radiation reactivity (Hsu et al., 2022). *Ex vivo* 3D organoid cultures derived from mouse jejunum and human ileum and colon were used to examine the radio-mitigative effects of CBP/P300 inhibitors (Rao et al., 2021).Transplanted colonic organoids were successfully transplanted onto the rectal mucosa of irradiated mice, reconstructing epithelial structure and integrity (Jee et al., 2021). Therefore, the development of organoids will contribute to the treatment of radiation enteritis.

### Microbial therapy of radiation enteritis

**FIGURE 5:**
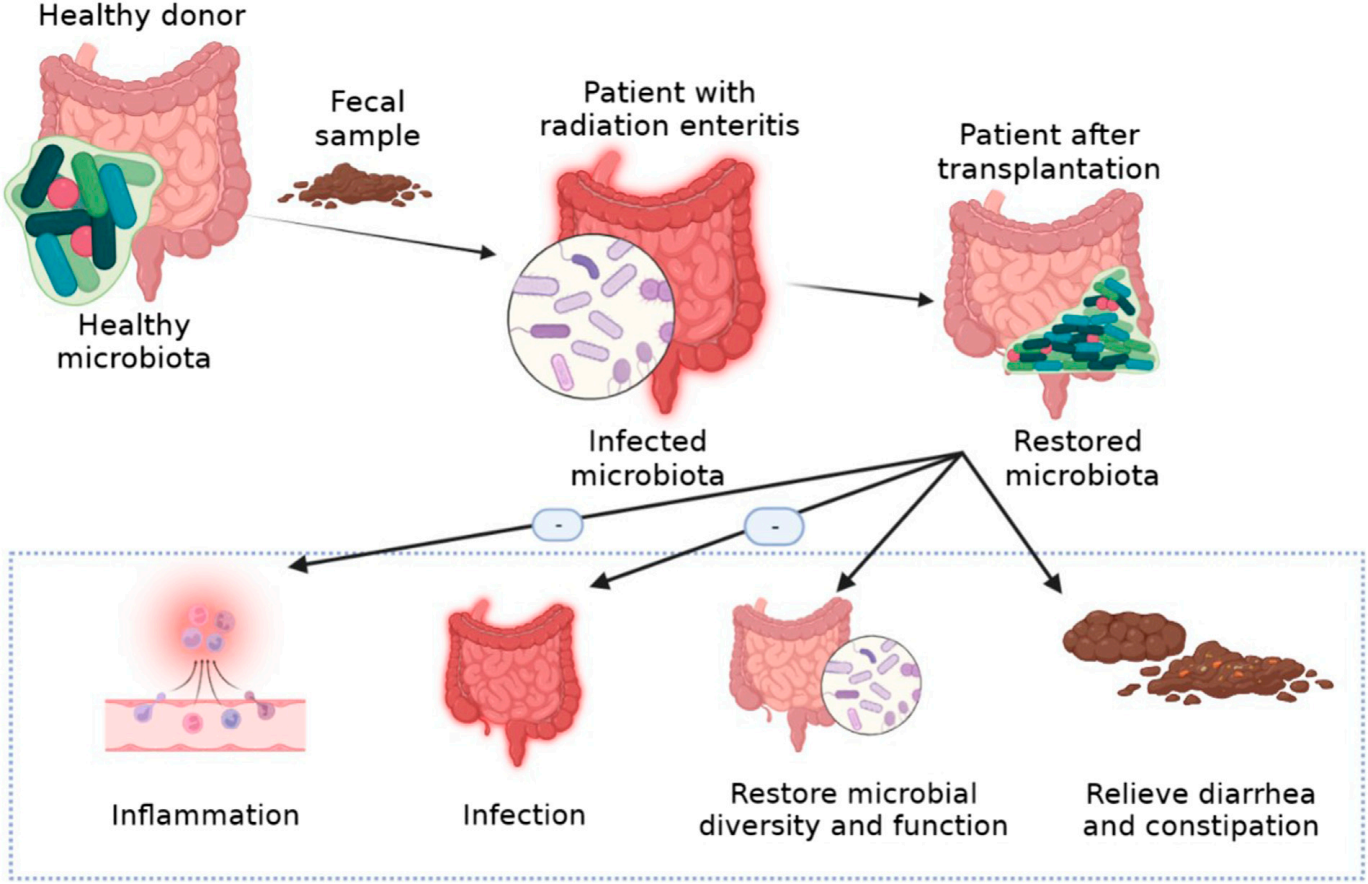
The treatment of FMT for radiation enteritis.

FMT is the administration of fecal matter from a donor into the intestinal tract of a recipient in order to directly change the recipient’s microbial composition and confer a health benefits (Gupta et al., 2016). FMT is a treatment method traditionally used for *Clostridium difficile* infections. However, it has since recently become an effective treatment for many diseases. It is a new strategy to treat diseases related to microbial imbalance by regulating intestinal flora (Figure 5). FMT has been shown to be effective in the treatment of type 2 diabetes mellitus, neurodegenerative diseases, autism spectrum disorder and so on (Wu et al., 2022; Matheson and Holsinger, 2023; Wang et al., 2023). FMT improved the irradiated animal survival rate, increased peripheral blood leukocyte count, and intestinal epithelial integrity, improved the gastrointestinal function, which can safely and effectively improve the symptoms of chronic radiation enteritis patients with intestinal and mucosa damage. FMT can also cause significant changes in the composition of intestinal bacteria and relieve the radiation symptoms of diarrhea and constipation (Ding et al., 2020). It has also been reported that FMT alleviates radiation toxicity without accelerating tumor growth by increasing the level of indole 3-propionic acid, a key gut microbial metabolite (Xiao et al., 2020). In addition, FMT is a potential treatment for radiation enteritis, which is characterized by intestinal flora disorder.

Probiotic therapy is a method to directly supplement probiotics to individuals with dysbiosis or probiotic deficiency to adjust the composition of the microbiome. It has been shown to achieve good results in a variety of treatments, such as Atopic dermatitis, seizures, kidney disease and metabolic diseases (Anania et al., 2022; Wang et al., 2022a; Dai et al., 2022). Probiotics have been studied to reduce radiation enteritis side effects by regulating the immune system, downregulating proinflammatory cytokines, regulating apoptosis and reversing ecological dysregulation (Linn et al., 2019). Studies have found that probiotic preparations increased the number of goblet cells and promoted the crypt cell proliferation, protected the intestinal epithelium, partly restored the diversity and composition of intestinal flora in irradiated mice (Zhao T. S. et al., 2021). In human patients, probiotic supplementation has been found to be potentially beneficial in the prevention and treatment of radiation-induced diarrhea (Hamad et al., 2013). Therefore, it is an effective strategy to adjust the microbiota structure for the treatment of radiation enteritis.

In addition to probiotics and fecal bacteria transplantation, drugs can also be combined with probiotics. For example, gavage *Lactobacillus reuteri* as a platform for the second-generation probiotic *Lactobacillus-reuteri*-Interleukin-22 (LR-IL-22) improved delivery of IL-22 to the irradiated intestine, and effectively reduced the intestinal damage caused by irradiated intestinal tract (Espinal et al., 2022).

Bacteriophages are one of the most abundant organisms in nature. They play an important role in maintaining the dynamics of human bacterial population and can effectively deal with and kill many infectious bacterial pathogens (Veeranarayanan et al., 2021). Therefore, it may be a feasible method to treat radiation-induced intestinal injury by affecting the microbiota structure by phage.

## Conclusion

The pathogenesis of radiation-induced intestinal injury is complex. However, in view of the lack of standard prevention and treatment methods for acute radiation-induced enteritis, the study of radiation-induced enteritis is particularly important. Stem cell therapy, organoids and FMT as the novel therapy all presented a certain effect on the treatment of radiation enteritis. FMT could directly target gut microbiota and reverse microbiota dysbiosis and intestinal inflammation in clinic, which are a promising therapy strategy for radiation enteritis.
